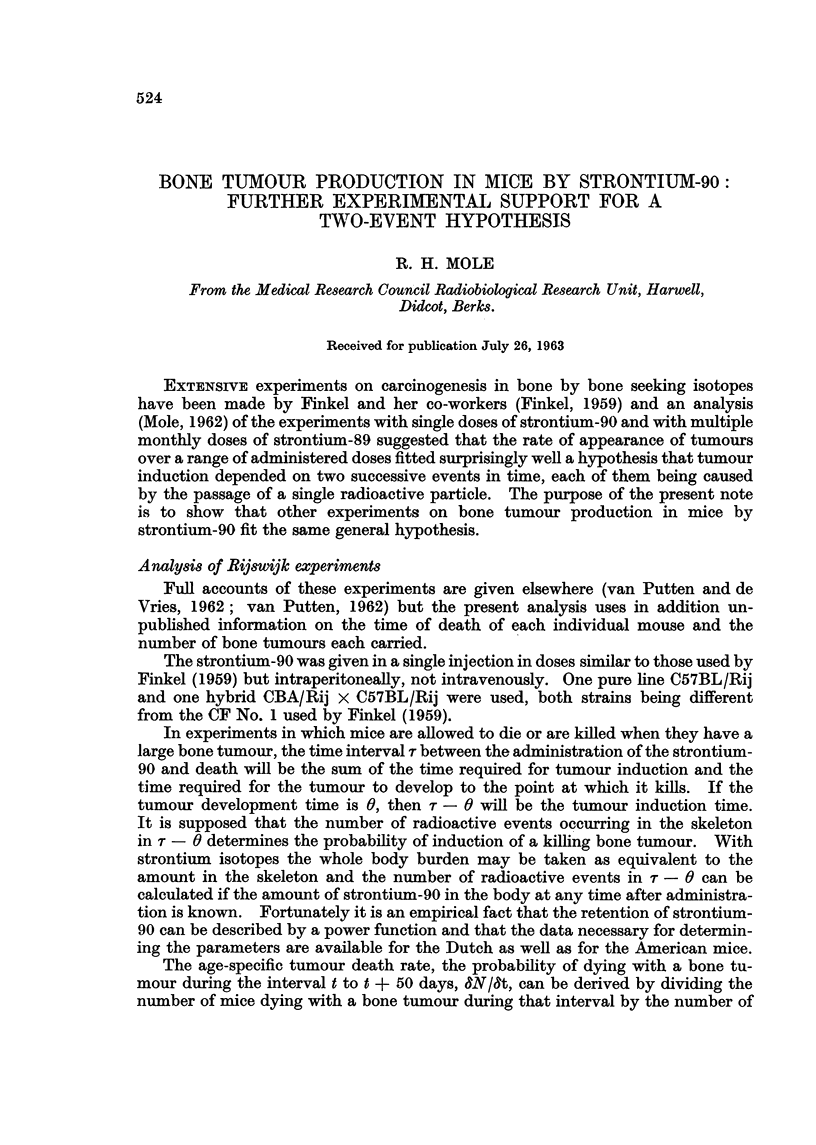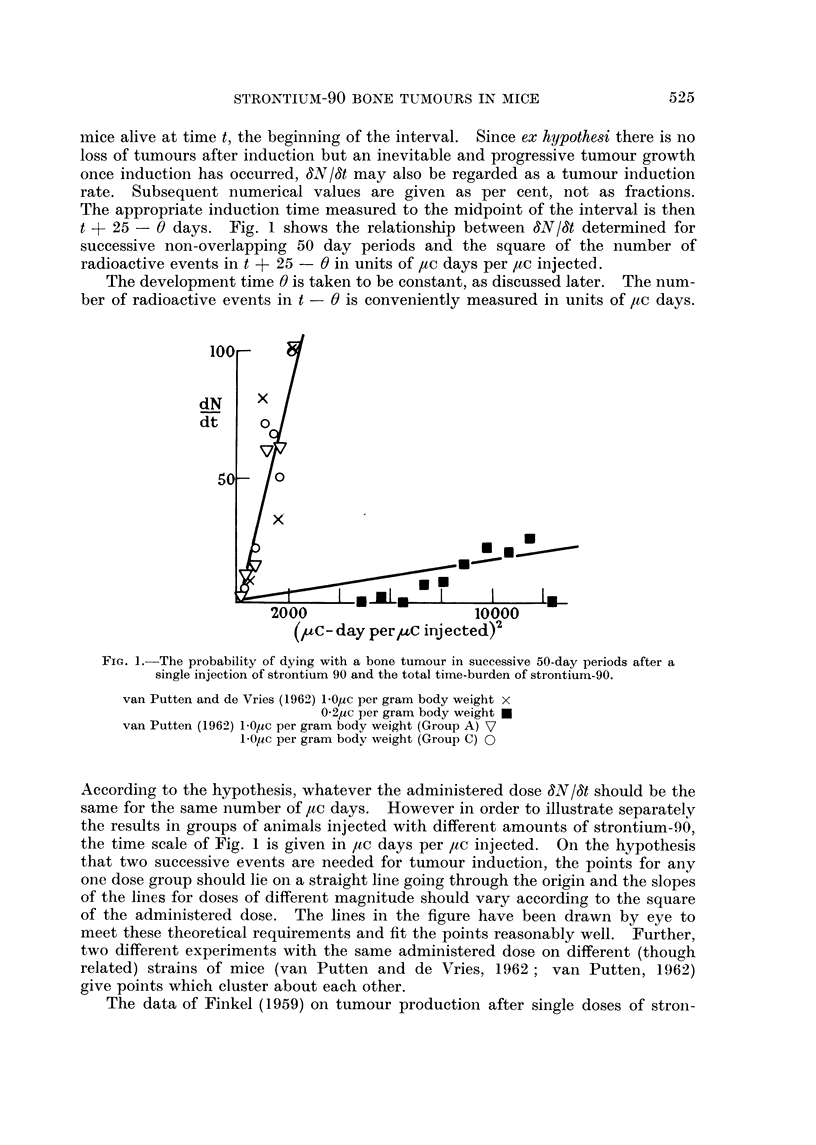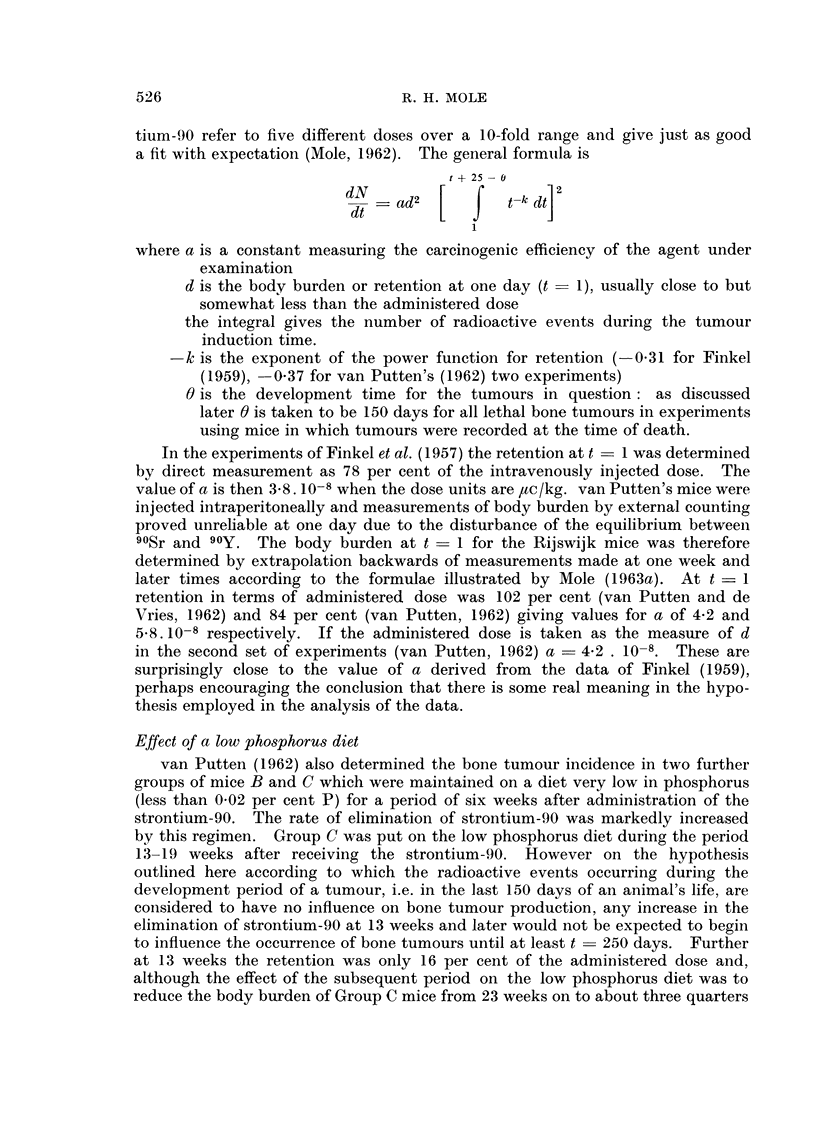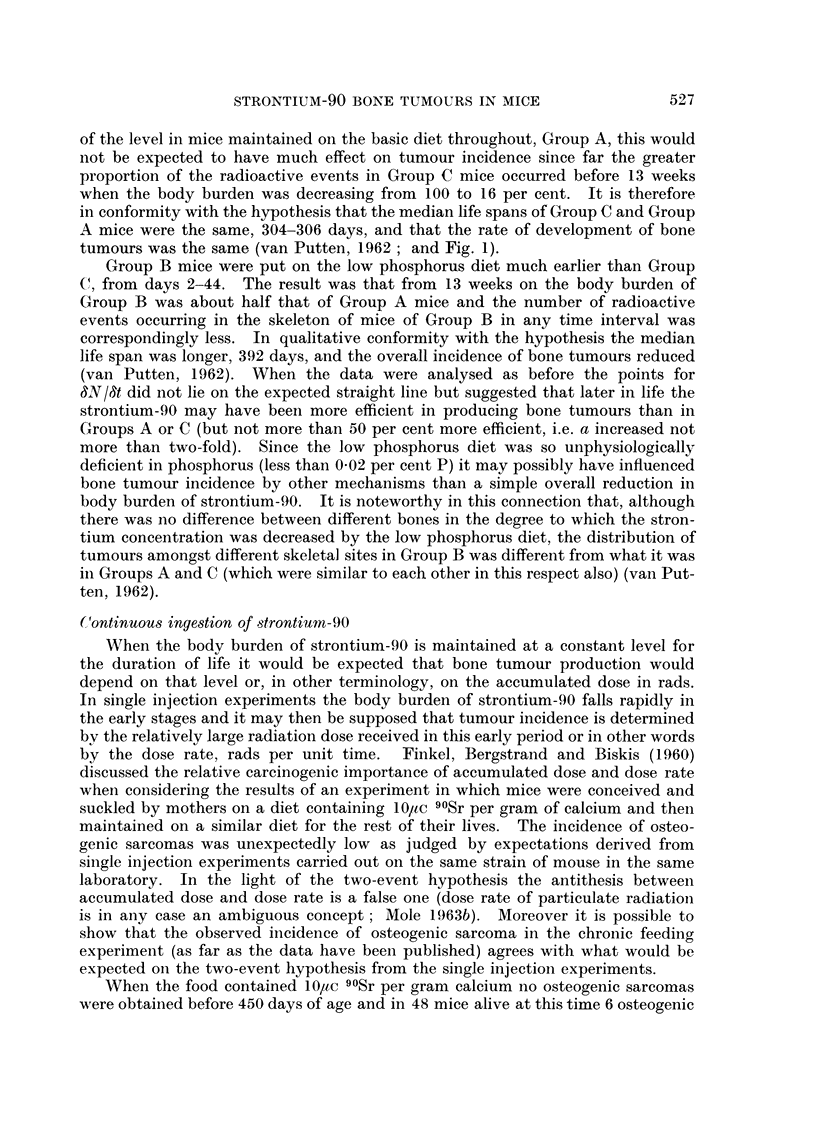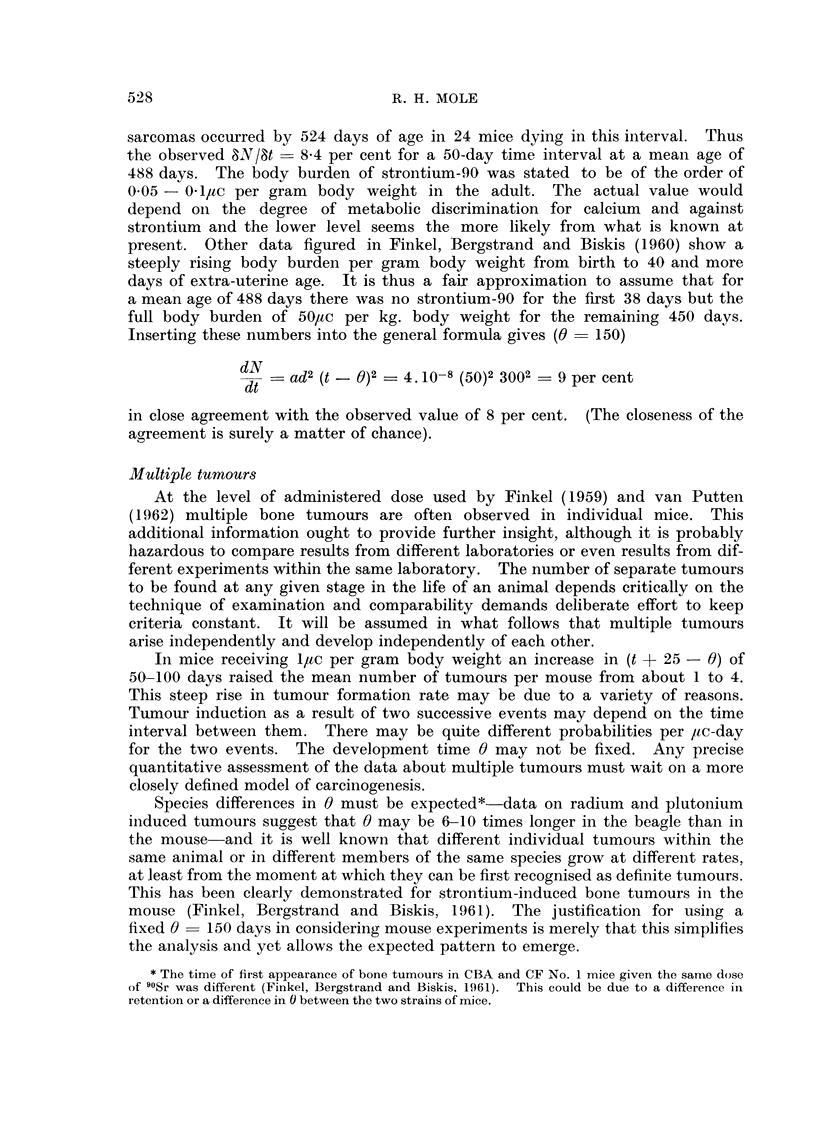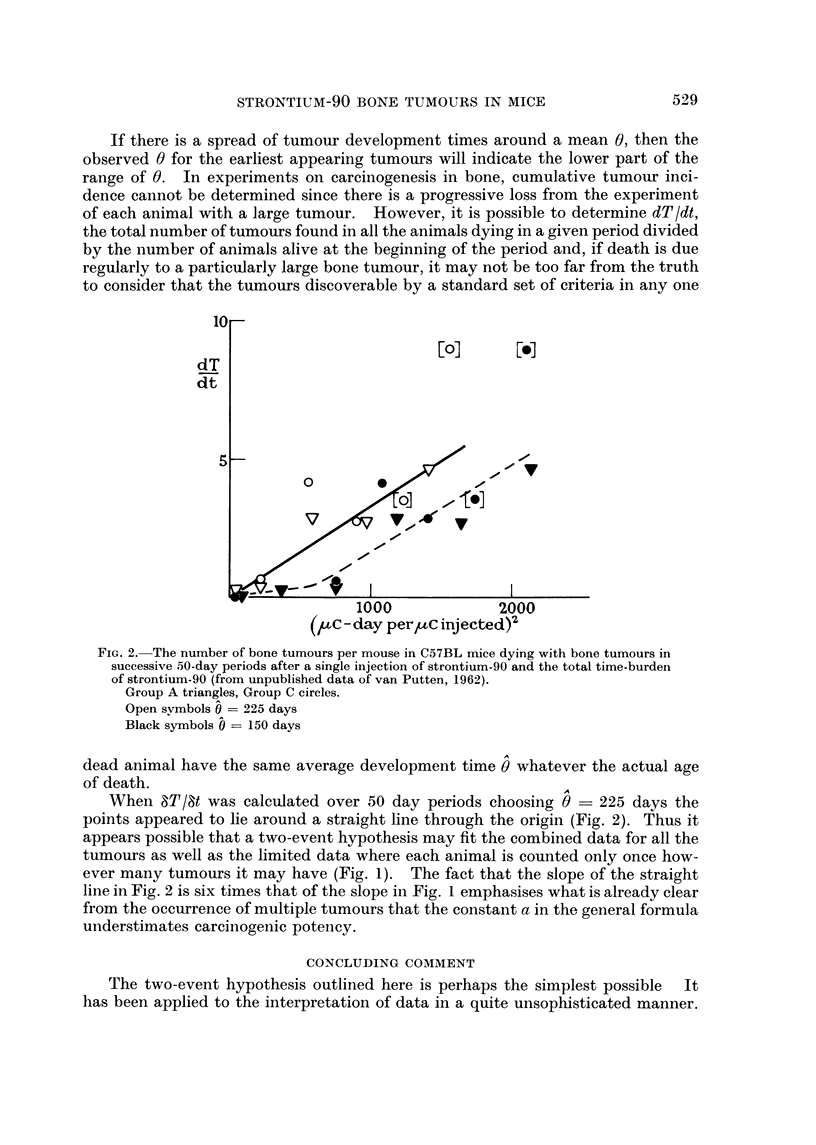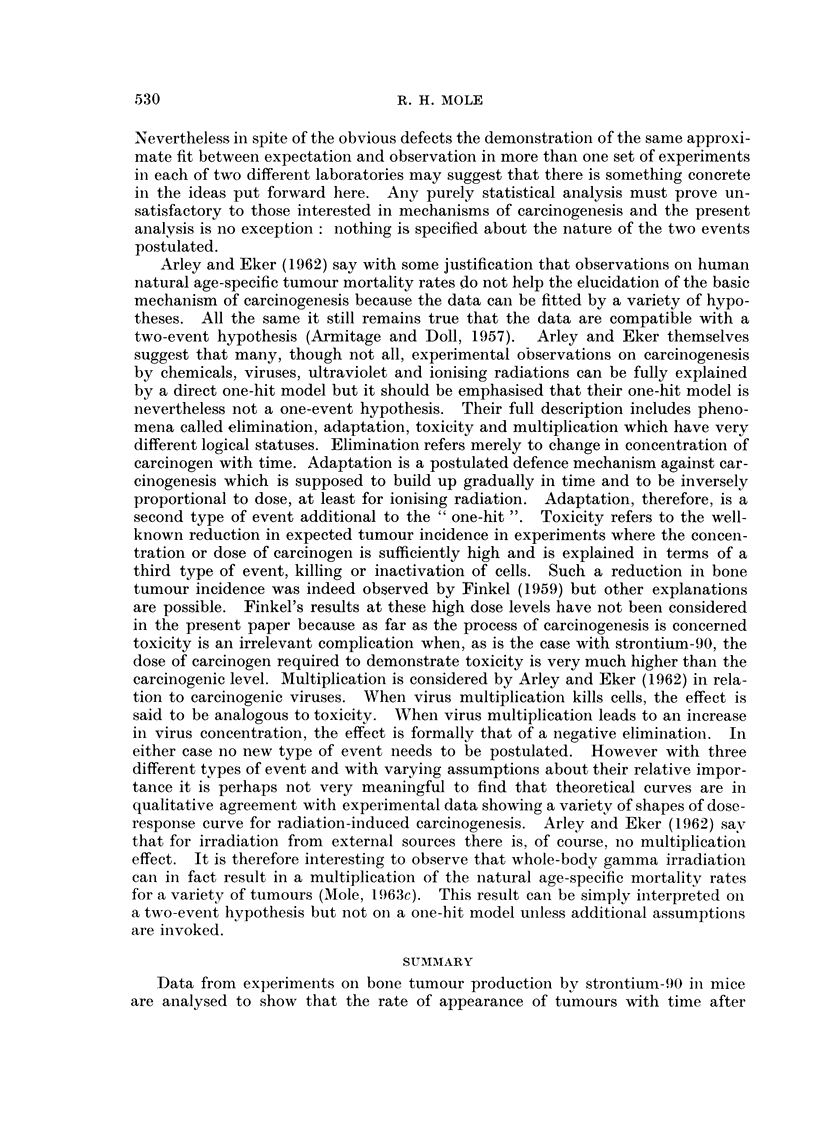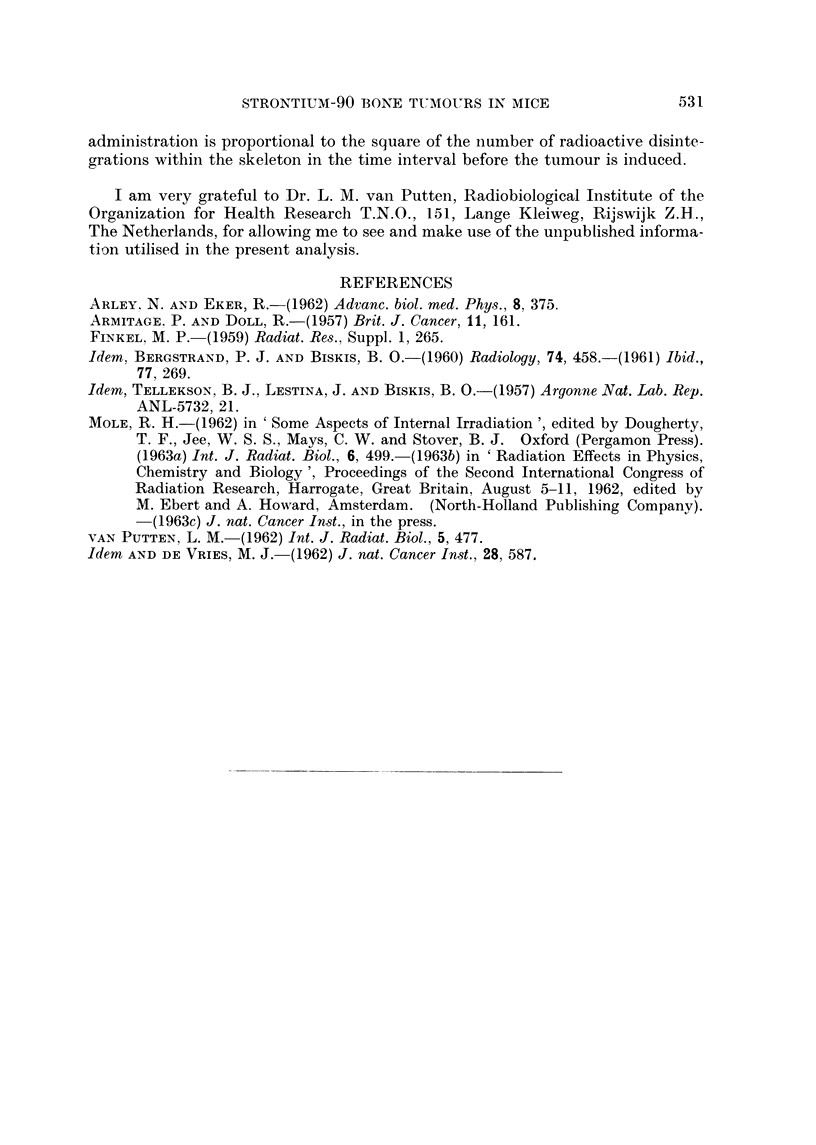# Bone Tumour Production in Mice by Strontium-90: Further Experimental Support for a Two-Event Hypothesis

**DOI:** 10.1038/bjc.1963.70

**Published:** 1963-09

**Authors:** R. H. Mole


### BONE TUMOUR PRODUCTION IN MICE BY STRONTIUM-90: FURTHER EXPERIMENTAL SUPPORT FOR A TWO-EVENT HYPOTHESIS

R. H. MOLE

*From the Medical Research Council Radiobiological Research Unit, Harwell, Didcot, Berks.*

Received for publication July 26, 1963

EXTENSIVE experiments on carcinogenesis in bone by bone seeking isotopes have been made by Finkel and her co-workers (Finkel, 1959) and an analysis (Mole, 1962) of the experiments with single doses of strontium-90 and with multiple monthly doses of strontium-89 suggested that the rate of appearance of tumours over a range of administered doses fitted surprisingly well a hypothesis that tumour induction depended on two successive events in time, each of them being caused by the passage of a single radioactive particle. The purpose of the present note is to show that other experiments on bone tumour production in mice by strontium-90 fit the same general hypothesis.

*Analysis of Rijswijk experiments*

Full accounts of these experiments are given elsewhere (van Putten and de Vries, 1962; van Putten, 1962) but the present analysis uses in addition unpublished information on the time of death of each individual mouse and the number of bone tumours each carried.

The strontium-90 was given in a single injection in doses similar to those used by Finkel (1959) but intraperitoneally, not intravenously. One pure line C57BL/Rij and one hybrid CBA/Rij $\times$ C57BL/Rij were used, both strains being different from the CF No. 1 used by Finkel (1959).

In experiments in which mice are allowed to die or are killed when they have a large bone tumour, the time interval $\tau$ between the administration of the strontium-90 and death will be the sum of the time required for tumour induction and the time required for the tumour to develop to the point at which it kills. If the tumour development time is $\theta$, then $\tau - \theta$ will be the tumour induction time. It is supposed that the number of radioactive events occurring in the skeleton in $\tau - \theta$ determines the probability of induction of a killing bone tumour. With strontium isotopes the whole body burden may be taken as equivalent to the amount in the skeleton and the number of radioactive events in $\tau - \theta$ can be calculated if the amount of strontium-90 in the body at any time after administration is known. Fortunately it is an empirical fact that the retention of strontium-90 can be described by a power function and that the data necessary for determining the parameters are available for the Dutch as well as for the American mice.

The age-specific tumour death rate, the probability of dying with a bone tumour during the interval $t$ to $t + 50$ days, $\delta N/\delta t$, can be derived by dividing the number of mice dying with a bone tumour during that interval by the number of mice alive at time $t$, the beginning of the interval. Since *ex hypothesi* there is no loss of tumours after induction but an inevitable and progressive tumour growth once induction has occurred, $\delta N/\delta t$ may also be regarded as a tumour induction rate. Subsequent numerical values are given as per cent, not as fractions. The appropriate induction time measured to the midpoint of the interval is then $t + 25 - \theta$ days. Fig. 1 shows the relationship between $\delta N/\delta t$ determined for successive non-overlapping 50 day periods and the square of the number of radioactive events in $t + 25 - \theta$ in units of $\mu$c days per $\mu$c injected.

The development time $\theta$ is taken to be constant, as discussed later. The number of radioactive events in $t - \theta$ is conveniently measured in units of $\mu$c days.

FIG. 1.—The probability of dying with a bone tumour in successive 50-day periods after a single injection of strontium 90 and the total time-burden of strontium-90.

van Putten and de Vries (1962) 1·0$\mu$c per gram body weight ×

0·2$\mu$c per gram body weight ■

van Putten (1962) 1·0$\mu$c per gram body weight (Group A) ▽

1·0$\mu$c per gram body weight (Group C) ○

According to the hypothesis, whatever the administered dose $\delta N/\delta t$ should be the same for the same number of $\mu$c days. However in order to illustrate separately the results in groups of animals injected with different amounts of strontium-90, the time scale of Fig. 1 is given in $\mu$c days per $\mu$c injected. On the hypothesis that two successive events are needed for tumour induction, the points for any one dose group should lie on a straight line going through the origin and the slopes of the lines for doses of different magnitude should vary according to the square of the administered dose. The lines in the figure have been drawn by eye to meet these theoretical requirements and fit the points reasonably well. Further, two different experiments with the same administered dose on different (though related) strains of mice (van Putten and de Vries, 1962 ; van Putten, 1962) give points which cluster about each other.

The data of Finkel (1959) on tumour production after single doses of strontium-90 refer to five different doses over a 10-fold range and give just as good a fit with expectation (Mole, 1962). The general formula is

$$\frac{dN}{dt} = ad^2 \left[ \int_1^{t + 25 - \theta} t^{-k} \, dt \right]^2$$

where $a$ is a constant measuring the carcinogenic efficiency of the agent under examination

$d$ is the body burden or retention at one day ($t = 1$), usually close to but somewhat less than the administered dose the integral gives the number of radioactive events during the tumour induction time.

$-k$ is the exponent of the power function for retention ($-0{\cdot}31$ for Finkel (1959), $-0{\cdot}37$ for van Putten's (1962) two experiments)

$\theta$ is the development time for the tumours in question : as discussed later $\theta$ is taken to be 150 days for all lethal bone tumours in experiments using mice in which tumours were recorded at the time of death.

In the experiments of Finkel *et al.* (1957) the retention at $t = 1$ was determined by direct measurement as 78 per cent of the intravenously injected dose. The value of $a$ is then $3{\cdot}8 \,.\, 10^{-8}$ when the dose units are $\mu$c/kg. van Putten's mice were injected intraperitoneally and measurements of body burden by external counting proved unreliable at one day due to the disturbance of the equilibrium between $^{90}$Sr and $^{90}$Y. The body burden at $t = 1$ for the Rijswijk mice was therefore determined by extrapolation backwards of measurements made at one week and later times according to the formulae illustrated by Mole (1963*a*). At $t = 1$ retention in terms of administered dose was 102 per cent (van Putten and de Vries, 1962) and 84 per cent (van Putten, 1962) giving values for $a$ of 4·2 and $5{\cdot}8 \,.\, 10^{-8}$ respectively. If the administered dose is taken as the measure of $d$ in the second set of experiments (van Putten, 1962) $a = 4{\cdot}2 \,.\, 10^{-8}$. These are surprisingly close to the value of $a$ derived from the data of Finkel (1959), perhaps encouraging the conclusion that there is some real meaning in the hypothesis employed in the analysis of the data.

*Effect of a low phosphorus diet*

van Putten (1962) also determined the bone tumour incidence in two further groups of mice $B$ and $C$ which were maintained on a diet very low in phosphorus (less than 0·02 per cent P) for a period of six weeks after administration of the strontium-90. The rate of elimination of strontium-90 was markedly increased by this regimen. Group $C$ was put on the low phosphorus diet during the period 13–19 weeks after receiving the strontium-90. However on the hypothesis outlined here according to which the radioactive events occurring during the development period of a tumour, i.e. in the last 150 days of an animal's life, are considered to have no influence on bone tumour production, any increase in the elimination of strontium-90 at 13 weeks and later would not be expected to begin to influence the occurrence of bone tumours until at least $t = 250$ days. Further at 13 weeks the retention was only 16 per cent of the administered dose and, although the effect of the subsequent period on the low phosphorus diet was to reduce the body burden of Group C mice from 23 weeks on to about three quarters of the level in mice maintained on the basic diet throughout, Group A, this would not be expected to have much effect on tumour incidence since far the greater proportion of the radioactive events in Group C mice occurred before 13 weeks when the body burden was decreasing from 100 to 16 per cent. It is therefore in conformity with the hypothesis that the median life spans of Group C and Group A mice were the same, 304–306 days, and that the rate of development of bone tumours was the same (van Putten, 1962 ; and Fig. 1).

Group B mice were put on the low phosphorus diet much earlier than Group C, from days 2–44. The result was that from 13 weeks on the body burden of Group B was about half that of Group A mice and the number of radioactive events occurring in the skeleton of mice of Group B in any time interval was correspondingly less. In qualitative conformity with the hypothesis the median life span was longer, 392 days, and the overall incidence of bone tumours reduced (van Putten, 1962). When the data were analysed as before the points for $\delta N/\delta t$ did not lie on the expected straight line but suggested that later in life the strontium-90 may have been more efficient in producing bone tumours than in Groups A or C (but not more than 50 per cent more efficient, i.e. $a$ increased not more than two-fold). Since the low phosphorus diet was so unphysiologically deficient in phosphorus (less than 0·02 per cent P) it may possibly have influenced bone tumour incidence by other mechanisms than a simple overall reduction in body burden of strontium-90. It is noteworthy in this connection that, although there was no difference between different bones in the degree to which the strontium concentration was decreased by the low phosphorus diet, the distribution of tumours amongst different skeletal sites in Group B was different from what it was in Groups A and C (which were similar to each other in this respect also) (van Putten, 1962).

*Continuous ingestion of strontium-90*

When the body burden of strontium-90 is maintained at a constant level for the duration of life it would be expected that bone tumour production would depend on that level or, in other terminology, on the accumulated dose in rads. In single injection experiments the body burden of strontium-90 falls rapidly in the early stages and it may then be supposed that tumour incidence is determined by the relatively large radiation dose received in this early period or in other words by the dose rate, rads per unit time. Finkel, Bergstrand and Biskis (1960) discussed the relative carcinogenic importance of accumulated dose and dose rate when considering the results of an experiment in which mice were conceived and suckled by mothers on a diet containing 10$\mu$c $^{90}$Sr per gram of calcium and then maintained on a similar diet for the rest of their lives. The incidence of osteogenic sarcomas was unexpectedly low as judged by expectations derived from single injection experiments carried out on the same strain of mouse in the same laboratory. In the light of the two-event hypothesis the antithesis between accumulated dose and dose rate is a false one (dose rate of particulate radiation is in any case an ambiguous concept ; Mole 1963*b*). Moreover it is possible to show that the observed incidence of osteogenic sarcoma in the chronic feeding experiment (as far as the data have been published) agrees with what would be expected on the two-event hypothesis from the single injection experiments.

When the food contained 10$\mu$c $^{90}$Sr per gram calcium no osteogenic sarcomas were obtained before 450 days of age and in 48 mice alive at this time 6 osteogenic sarcomas occurred by 524 days of age in 24 mice dying in this interval. Thus the observed $\delta N/\delta t = 8{\cdot}4$ per cent for a 50-day time interval at a mean age of 488 days. The body burden of strontium-90 was stated to be of the order of $0{\cdot}05 - 0{\cdot}1\mu c$ per gram body weight in the adult. The actual value would depend on the degree of metabolic discrimination for calcium and against strontium and the lower level seems the more likely from what is known at present. Other data figured in Finkel, Bergstrand and Biskis (1960) show a steeply rising body burden per gram body weight from birth to 40 and more days of extra-uterine age. It is thus a fair approximation to assume that for a mean age of 488 days there was no strontium-90 for the first 38 days but the full body burden of $50\mu c$ per kg. body weight for the remaining 450 days. Inserting these numbers into the general formula gives ($\theta = 150$)

$$\frac{dN}{dt} = ad^2\,(t - \theta)^2 = 4.10^{-8}\,(50)^2\,300^2 = 9 \text{ per cent}$$

in close agreement with the observed value of 8 per cent. (The closeness of the agreement is surely a matter of chance).

*Multiple tumours*

At the level of administered dose used by Finkel (1959) and van Putten (1962) multiple bone tumours are often observed in individual mice. This additional information ought to provide further insight, although it is probably hazardous to compare results from different laboratories or even results from different experiments within the same laboratory. The number of separate tumours to be found at any given stage in the life of an animal depends critically on the technique of examination and comparability demands deliberate effort to keep criteria constant. It will be assumed in what follows that multiple tumours arise independently and develop independently of each other.

In mice receiving $1\mu c$ per gram body weight an increase in $(t + 25 - \theta)$ of 50–100 days raised the mean number of tumours per mouse from about 1 to 4. This steep rise in tumour formation rate may be due to a variety of reasons. Tumour induction as a result of two successive events may depend on the time interval between them. There may be quite different probabilities per $\mu c$-day for the two events. The development time $\theta$ may not be fixed. Any precise quantitative assessment of the data about multiple tumours must wait on a more closely defined model of carcinogenesis.

Species differences in $\theta$ must be expected*—data on radium and plutonium induced tumours suggest that $\theta$ may be 6–10 times longer in the beagle than in the mouse—and it is well known that different individual tumours within the same animal or in different members of the same species grow at different rates, at least from the moment at which they can be first recognised as definite tumours. This has been clearly demonstrated for strontium-induced bone tumours in the mouse (Finkel, Bergstrand and Biskis, 1961). The justification for using a fixed $\theta = 150$ days in considering mouse experiments is merely that this simplifies the analysis and yet allows the expected pattern to emerge.

* The time of first appearance of bone tumours in CBA and CF No. 1 mice given the same dose of $^{90}Sr$ was different (Finkel, Bergstrand and Biskis, 1961). This could be due to a difference in retention or a difference in $\theta$ between the two strains of mice.

If there is a spread of tumour development times around a mean $\theta$, then the observed $\theta$ for the earliest appearing tumours will indicate the lower part of the range of $\theta$. In experiments on carcinogenesis in bone, cumulative tumour incidence cannot be determined since there is a progressive loss from the experiment of each animal with a large tumour. However, it is possible to determine $dT/dt$, the total number of tumours found in all the animals dying in a given period divided by the number of animals alive at the beginning of the period and, if death is due regularly to a particularly large bone tumour, it may not be too far from the truth to consider that the tumours discoverable by a standard set of criteria in any one

FIG. 2.—The number of bone tumours per mouse in C57BL mice dying with bone tumours in successive 50-day periods after a single injection of strontium-90 and the total time-burden of strontium-90 (from unpublished data of van Putten, 1962).
Group A triangles, Group C circles.
Open symbols $\hat{\theta}$ = 225 days
Black symbols $\hat{\theta}$ = 150 days dead animal have the same average development time $\hat{\theta}$ whatever the actual age of death.

When $\delta T/\delta t$ was calculated over 50 day periods choosing $\hat{\theta}$ = 225 days the points appeared to lie around a straight line through the origin (Fig. 2). Thus it appears possible that a two-event hypothesis may fit the combined data for all the tumours as well as the limited data where each animal is counted only once however many tumours it may have (Fig. 1). The fact that the slope of the straight line in Fig. 2 is six times that of the slope in Fig. 1 emphasises what is already clear from the occurrence of multiple tumours that the constant $a$ in the general formula understimates carcinogenic potency.

### CONCLUDING COMMENT

The two-event hypothesis outlined here is perhaps the simplest possible It has been applied to the interpretation of data in a quite unsophisticated manner.

Nevertheless in spite of the obvious defects the demonstration of the same approximate fit between expectation and observation in more than one set of experiments in each of two different laboratories may suggest that there is something concrete in the ideas put forward here. Any purely statistical analysis must prove unsatisfactory to those interested in mechanisms of carcinogenesis and the present analysis is no exception : nothing is specified about the nature of the two events postulated.

Arley and Eker (1962) say with some justification that observations on human natural age-specific tumour mortality rates do not help the elucidation of the basic mechanism of carcinogenesis because the data can be fitted by a variety of hypotheses. All the same it still remains true that the data are compatible with a two-event hypothesis (Armitage and Doll, 1957). Arley and Eker themselves suggest that many, though not all, experimental observations on carcinogenesis by chemicals, viruses, ultraviolet and ionising radiations can be fully explained by a direct one-hit model but it should be emphasised that their one-hit model is nevertheless not a one-event hypothesis. Their full description includes phenomena called elimination, adaptation, toxicity and multiplication which have very different logical statuses. Elimination refers merely to change in concentration of carcinogen with time. Adaptation is a postulated defence mechanism against carcinogenesis which is supposed to build up gradually in time and to be inversely proportional to dose, at least for ionising radiation. Adaptation, therefore, is a second type of event additional to the " one-hit ". Toxicity refers to the well-known reduction in expected tumour incidence in experiments where the concentration or dose of carcinogen is sufficiently high and is explained in terms of a third type of event, killing or inactivation of cells. Such a reduction in bone tumour incidence was indeed observed by Finkel (1959) but other explanations are possible. Finkel's results at these high dose levels have not been considered in the present paper because as far as the process of carcinogenesis is concerned toxicity is an irrelevant complication when, as is the case with strontium-90, the dose of carcinogen required to demonstrate toxicity is very much higher than the carcinogenic level. Multiplication is considered by Arley and Eker (1962) in relation to carcinogenic viruses. When virus multiplication kills cells, the effect is said to be analogous to toxicity. When virus multiplication leads to an increase in virus concentration, the effect is formally that of a negative elimination. In either case no new type of event needs to be postulated. However with three different types of event and with varying assumptions about their relative importance it is perhaps not very meaningful to find that theoretical curves are in qualitative agreement with experimental data showing a variety of shapes of dose-response curve for radiation-induced carcinogenesis. Arley and Eker (1962) say that for irradiation from external sources there is, of course, no multiplication effect. It is therefore interesting to observe that whole-body gamma irradiation can in fact result in a multiplication of the natural age-specific mortality rates for a variety of tumours (Mole, 1963*c*). This result can be simply interpreted on a two-event hypothesis but not on a one-hit model unless additional assumptions are invoked.

### SUMMARY

Data from experiments on bone tumour production by strontium-90 in mice are analysed to show that the rate of appearance of tumours with time after administration is proportional to the square of the number of radioactive disintegrations within the skeleton in the time interval before the tumour is induced.

I am very grateful to Dr. L. M. van Putten, Radiobiological Institute of the Organization for Health Research T.N.O., 151, Lange Kleiweg, Rijswijk Z.H., The Netherlands, for allowing me to see and make use of the unpublished information utilised in the present analysis.

### REFERENCES

ARLEY, N. AND EKER, R.—(1962) *Advanc. biol. med. Phys.*, **8**, 375.

ARMITAGE, P. AND DOLL, R.—(1957) *Brit. J. Cancer*, **11**, 161.

FINKEL, M. P.—(1959) *Radiat. Res.*, Suppl. 1, 265.

*Idem*, BERGSTRAND, P. J. AND BISKIS, B. O.—(1960) *Radiology*, **74**, 458.—(1961) *Ibid.*, **77**, 269.

*Idem*, TELLEKSON, B. J., LESTINA, J. AND BISKIS, B. O.—(1957) *Argonne Nat. Lab. Rep.* ANL-5732, 21.

MOLE, R. H.—(1962) in ‘ Some Aspects of Internal Irradiation ’, edited by Dougherty, T. F., Jee, W. S. S., Mays, C. W. and Stover, B. J. Oxford (Pergamon Press). (1963*a*) *Int. J. Radiat. Biol.*, **6**, 499.—(1963*b*) in ‘ Radiation Effects in Physics, Chemistry and Biology ’, Proceedings of the Second International Congress of Radiation Research, Harrogate, Great Britain, August 5–11, 1962, edited by M. Ebert and A. Howard, Amsterdam. (North-Holland Publishing Company). —(1963*c*) *J. nat. Cancer Inst.*, in the press.

VAN PUTTEN, L. M.—(1962) *Int. J. Radiat. Biol.*, **5**, 477.

*Idem* AND DE VRIES, M. J.—(1962) *J. nat. Cancer Inst.*, **28**, 587.